# Case of Obscure Cerebral Disease Following Injury

**Published:** 1862-02

**Authors:** H. P. Babcock

**Affiliations:** Medical Student


ART. III.—*Case of Obscure Cerebral disease following injury. Communicated to* PROF. ROCHESTER *by* IRA D. HOPKINS, M. D., *of Utica.—Arranged and condensed by* H. P. BABCOCK *Medical Student.*

July 19, '60—Patient was bathing with some friends and immediately on rising to the surface after diving, complained of having struck his head against the bottom. Vertigo ensued, but no other unpleasant symptoms, he continued in his business until the 22nd. The following symptoms were then manifest: Severe headache, skin hot and dry, eyes suffused, pupils contracted, sensibility to light and sound acute, tongue red, dry and pointed, emesis, constipation.

℞ Hydrag. chl. mite grs. ij. Ol. Tiglii. gtt. iij. Elaterii gr. ¼. M. ft. pil. Also ℞ extr. conii et hydrag. chl. mite. a a gr. i. Camphoræ gr. ½. M. every 4 hours.

Sinipisim to nape of neck and feet. Ice to head. Diet light.

23d.—No sleep; two dejections; pain in head intense; nausea and vomiting; pulse hard and frequent; skin more moist; respiration accelerated and labored; convulsive movements of left side; excessive thirst; urine high colored and scanty; other symptoms same. C. T.

24th.—Had about two hours sleep; bad dreams; nausea increased; constipation continues; tongue white, moist and very pointed; increase of convulsive movements; passive delirium.

℞ Hyosciami gr. 1. Hydrag. chl. mite et Camphoræ a a gr. ½. M. ft. pil. One every two hours; continue siapisms and ice; diet same and enema of Syrup ℥ii. Magnes. Sulph. ℥iv. Aquæ.

Oj. M. Dejection small and scybalous. At night Elix. McMunn gtts. x x v.

25th.—General symptoms same; pain extending to neck; countenance anxious; continue pil of hyoseami, &c., and enema of Syrup ℥ij. Fl. extr. rhei et sennæ ℥iv. Magnes. sulph. ℥ij. Aquæ. Oiss M. Movement same as before.

26th.—More sleep; symptoms same; less thirst.

℞ Morph. sulph. gr. $\frac{1}{7}$. Hydr. chl. mite gr. $\frac{3}{4}$. Camph. gr. $\frac{1}{2}$. Quiniæ sulph. gr. $\frac{1}{8}$. M. ft. pil—one every four hours.

Blister to back of neck; continue sinapisms and ice; also enema.

27th.—Intense pain in head and neck continues; tongue more coated; pulse less hard and frequent; other symptoms same; continue pills and sinapisms as yesterday; also

℞ Fl. extr. rhei et sennæ ʒiv. Hydr. chl. mite grs. viij. M. Diet light with iced milk.

28th.—Less pain; sense of light and sound very acute; bowels more natural; pulse 80, and soft; pupils less contracted; Drs. Coventry and Thomas in consultation.

℞ Morph. sulph. gr. $\frac{1}{8}$. Hydr. chl. mite gr. $\frac{3}{4}$. Camph. and Ipecac ā ā gr. $\frac{1}{2}$. M. ft. pil.; one every four hours, alternating with Ammon. carb. grs. iij.; continue ice and sinapisms; diet light.

29th.—Increase of pain; pupils widely dilated; very sensitive to light; nausea increased; rather stupid; bowels natural; slight strangury; continue pills of yesterday every six hours; ammon. carb. and sinapisms; whisky sling; bathe body in spirits.

30th.—Symptoms same; more stupid; stop pills and give brandy ʒij every four hours, alternating with ammon. carb.

31st.—Passed a good night; still complains of pain in head, but more tolerance of light and sound; twitching of the eyes; tongue clearing off; Drs. Coventry and Thomas again in consultation; treatment same.

August 1st.—Same.

2d.—Had considerable sleep; pain continues; tolerance of light and sound; bowels natural; nausea ceased; urine lighter colored and albuminous; partial hemiplegia of left side; continue as before with tannin gr. $\frac{1}{2}$ in brandy; better diet.

3d.—Patient much improved; countenance bright; during the day bled from right ear about ℥ss; continue brandy and ammon. carb., also quiniae. sulph. gr. $\frac{1}{4}$.

4th.—Much better; sat up. ℞ brandy ʒij.; quiniae. sulph. gr. j.; M. every six hours.

5th to 8th.—Improvement steady; light headed. C. T.

9th.—Considerable better, but complains of head; numbness in fingers and toes of left side; vision and articulation impaired; pulse 70; rode out. C. T.

10th and 11th.—Symptoms more favorable, but he was drowsy, and had occasional *stupid turns*, each lasting about an hour.

12th.—Still complains of a "queer sensation in the head;" pain in back of neck, and over præcordium, accompanied by a sense of oppression in the chest; this latter pain has troubled him for years, when indisposed; physical exploration disclosed nothing abnormal in thorax. Emp. picis. et belladon. on side.

13th to 16th.—The stupid turns recur at intervals; urine not albuminous; continue quiniae sulph. gr. i.; stop brandy,

17th to 25th.—Improvement gradual.—C. T. Has had meat, &c. since 10th; cessation of pain.

26th.—℞ Quiniae. sulph. grs. ij. Bis die, and on 29th discharged "quite smart."

